# On Randolph Blake

**DOI:** 10.1167/jov.26.10.21

**Published:** 2026-10-21

**Authors:** Sam Ling, Jan W. Brascamp, Frank Tong

**Affiliations:** 1 Department of Psychological & Brain Sciences, Boston University, Boston, MA, USA; 2 Department of Psychology, Michigan State University, East Lansing, MI, USA; 3 Department of Psychology and Vanderbilt Vision Research Center, Vanderbilt University, Nashville, TN, USA

**Keywords:** binocular rivalry, psychophysics, visual awareness, mentorship, history of vision science

## Abstract

Randolph Blake has had a profound influence on the field of vision science. In a career spanning over five decades, he has revealed time and again the magic of psychophysics, leveraging it to make major contributions to our understanding of basic vision, rivalry, biological motion perception, multisensory processing, synesthesia, and the neural bases of visual awareness, to name a few examples. He has also mentored countless graduate students and postdoctoral researchers, many of whom have gone on to shape vision science in their own right. In this retrospective, we honor Randolph's legacy as a scientist and as a mentor. Rather than aiming for an exhaustive survey of his corpus of work, we have instead tried to paint a portrait of what we consider an aspirational career in science: one that valued process over product and mentorship over metrics. A constant throughout Randolph's career was something hard to quantify: a mind characterized by a knack for creative experimental design and by a persistent curiosity that led him to pursue vision science's most important unanswered questions. We hope this piece captures not only his scientific contributions but also the personality he brought to his academic roles and how that shaped the people fortunate enough to work alongside him.

## The scientist and the science

This should sound familiar to anyone who has had the privilege of working alongside Randolph Blake: You've collected your experimental data, you've explored seemingly every corner of possible analyses, you've scoured the literature for any glimmer of insight, and you're at a loss as to what to make of it all. So, you walk into Randolph's office; he drops whatever he's working on at that moment to come sit with you, and you spend the next hour or so exploring the state space together. And then, some sort of wizardry happens. Because without fail, when you walk out of his office, you're left with a sense of stunned delight: How did we just turn what felt like a failed set of results into a fresh finding, suddenly alive, with exciting follow-up analyses and experiments?

If you haven't worked with Randolph, this may sound dramatic, or like an account of an exceptional but isolated interaction. But if you have, you know: This sort of thing happened all the time. Working with Randolph was like that. Whether your conundrum came from an unruly dataset, an impossible experiment, or a skeptical reviewer, with Randolph on board, there was a perspective that resolved it—it was just a matter of finding it. No matter how long it's been since you last sat there in his office, hunched over a laptop or a pile of papers, those moments are likely among your best memories as a researcher. Because in his lab, you got the sense that while the products of science mattered, the *process* was where the real joy lived. Both that exceptional ability and that joy are reflected in a career of remarkable productivity: over 300 published papers, more than 35,000 citations, and four dozen trained scientists carrying that approach forward in their own labs. In this retrospective, we foreground both of those legacies: the science and the scientist who made doing research feel so fun, you'd forget it was actual work.

 

**Lee Gilroy:** Working with Randolph was both humbling and inspiring. Imagine being on a team with Michael Jordan or Wayne Gretzky—that's what it felt like. Coauthoring papers with Randolph and his writing talent was inspirational—plus I learned how to incorporate woodworking terms like “dovetail” and “orthogonal” into my writing!

Randolph Blake's research interests have been wide-reaching—in part driven by his intellectual curiosity, but also a testament to his ability to find common ground with the diverse range of perspectives in his trainees and collaborators. Over the course of his career, he has made fundamental contributions to our understanding of binocular vision, stereopsis and rivalry, visual adaptation, motion processing, biological motion perception, center–surround interactions, multisensory integration, synesthesia, and the neural bases of visual awareness. What's striking is not just the breadth of that list but the coherence beneath it: Each thread reflects a restless curiosity about how the visual system constructs a stable, meaningful representation of the world from ambiguous and ever-changing inputs. This balance of coherence and intellectual curiosity is directly reflected in this special issue honoring the contributions of Randolph Blake, which covers *rivalry and multistable perception* (Alais & Kim, 2025; Bressler, Noah, Lu, & Silver, 2026; Cardoso, Li, Heeger, & Dugué, 2025; Coorey, Davidson, & Alais, 2026; Huang, Jiang, & Meng, 2025; Whyte, Wilson, Shine, & Alais, 2026 Zhang, Marshev, & Brascamp, 2025), *attention and visual search* (Huszar, Vizel, & Carrasco, 2025; Lappin & Bell, 2026; Lee & Carrasco, 2026), *neuroimaging of the visual system* (Hong & Tong, 2025; Vinke, Ling, & Bloem, 2026), *biological motion* (Swann, Davidson, Clouston, & Alais, 2025), *visual illusions* (Riga, Anstis, Thornton, & Cavanagh, 2025), *aging* (Norman, Graham, & Eaton, 2025), *multisensory processing* (Dai & Hsieh, 2025; Song, Shin, & Kim, 2025), and *basic visual function* (Basim, Goddard, Yang, & Webster, 2025; Paffen, 2025).

A look at Randolph's publications shows the work of a psychophysicist at heart. Certainly, he brought a flexibly expanding toolkit to bear on his questions—functional magnetic resonance imaging (fMRI), computational modeling, transcranial magnetic stimulation, and the study of visual processing in clinical populations. Nevertheless, regardless of the phenomenon under question or the method being utilized, Randolph's work has revealed time and again the extraordinary power of the *psychophysical approach*—its facility for precisely carving nature at its seams. Rather than solving problems through a technological show of force, Randolph has had a preternatural talent for devising elegant solutions drawn primarily from the psychophysical toolkit, only turning to other methods when they were truly necessary to answer the question at hand. Take neuroimaging or electrophysiology: Informative and technically impressive, and Randolph's career includes important contributions using both. But his work consistently reminds us that visual system function cannot be fully apprehended without a deep understanding of perception itself and of the critical elements that give rise to what we actually see. Survey the creative expanse of his published work and one conclusion becomes difficult to escape: *Visual* *psychophysics is not merely one tool among many*. It's a scientific philosophy that lies at the heart of vision science, providing the keys to unlocking the mysteries of visual perception using whatever methodological approach the mystery at hand calls for. That conviction, held with both confidence and humility, shaped everything about how Randolph approached a research problem—and, as we will see, how he taught others to approach problems.

 

**Sang Chul Chong:** Randolph has immense curiosity. Who else would try ox knee soup on their first attempt, let alone ask for the recipe without knowing the language?

 

**Robert O'Shea:** When I arrived for my Northwestern postdoctoral fellowship with Randolph in 1985, he picked me up from O'Hare, drove me to his beautiful house in Evanston, and ushered me into my own bedroom for the next few days. Randolph made me welcome into his family life with Elaine, feeding me, even watching TV with me one night. He also found an apartment for me, just north of Northwestern's stadium, where I settled in. Randolph introduced me to his academic family at work with whom I made lifelong friendships. I thought Randolph was lucky to have such a blessed group of academic children. All of this allowed Randolph and me to quickly start doing good science together. Much later, after Randolph moved to Vanderbilt, I visited him for about 10 days to tackle a scientific problem we had discussed. Randolph provided the same treatment as at Northwestern, this time in the beautiful house he and Elaine built. At work, I found a brand-new set of blessed academic children—the only constant was that they were chosen and mentored by Randolph. He also found a way to solve what seemed to me, after days staring into his stereoscope at probe stimuli flashing briefly on rivalry displays, to be an intractable scientific problem. Brilliant! Randolph has a way of making the lives of those he touches fun (e.g., softball, bicycle riding, Chicago pizza, music) and of getting the best out of those he touches at work (i.e., good fun science). He is gentle, supportive, smart, creative, and hard-working. I am honored he mentored me.

## With the back of the brain in the back of the mind

Randolph's work has always sat at the intersection of psychology and biology. This can be seen early on in his PhD studies with Robert Fox (Vanderbilt University), where he trained in a kind of psychology that treats perception as fundamentally a biological phenomenon. Around the time he was doing his PhD and postdoctoral work (1968–1974), vision science was experiencing a sea change, ushered in by a wave of electrophysiological discoveries, which were being carried out largely in cats (Barlow, Blakemore, & Pettigrew, 1967; Blakemore & Cooper, 1970; Enroth-Cugell & Robson, 1966; Hubel & Wiesel, 1959; Hubel & Wiesel, 1962; Hubel & Wiesel, 1970). Randolph was one of the pioneers at the time who intuited the importance of leveraging rigorous psychophysics to bridge behavior with this growing body of single-unit studies. This scientific intuition proved not only prescient but also immediately consequential, as his PhD and postdoctoral years alone yielded three papers in *Nature*!

 

**Karen Holopgian:** My research in the Randolph Blake lab started in the vision labs at Northwestern University. My early work focused on cat psychophysics, and somewhat fortuitously, we did a project on visual function in strabismic cats. This sparked my interest in clinical questions, and I eventually did my dissertation on strabismus and amblyopia in humans. Following this, I spent >20 years studying patients with retinal degenerations in the Ophthalmology Department of NYU Medical Center and currently run clinical trials in Ophthalmology for Novartis Pharmaceutical Company. So, I thank Randolph for my first research project on strabismus, which started my lifelong research in clinical ophthalmology. I also thank him for being an amazing mentor, someone who encouraged me to think independently, to trust my instincts, and to love the science! He was incredibly supportive, as well as hard-working, brilliant, and, last but not least, ensured the work was fun and rewarding! Kudos to a great scientist!

In his first *Nature* paper, Randolph developed a novel behavioral paradigm to train cats to report their perceptions of stereoscopic depth, establishing their ability to perceive stereopsis (Fox & Blake, 1971). This paradigm laid the foundation for many subsequent studies, including work on the impact of alternating monocular deprivation on binocular depth perception in cats (Blake & Hirsch, 1975) and the characterization of strabismus and impaired visual resolution in cats (Blake & Crawford, 1974; Blake & Antoinetti, 1976).

While the bulk of Randolph's early research output concerns human vision, rather than cat vision, a look at any part of his impressive body of work reveals a researcher who consistently held perception's biological origins in the back of his mind. One case in point is Randolph's recurring use of what he refers to as *psychoanatomical methods*: an approach that centers on participant reports in carefully designed experimental settings, with the aim of noninvasively inferring and characterizing steps in the processing sequence from sensation to perception to behavior. Randolph's second article in *Nature* (Blake & Fox, 1972) exemplifies this approach. In this study, they established that contrast adaptation to oriented gratings depends on binocular cortical mechanisms. Specifically, they discovered that monocular adaptation of one eye leads to reliable interocular transfer effects when the test stimulus is subsequently presented to the fellow eye. Moreover, this interocular transfer was observed even after temporary pressure-blinding of the adapted eye, thereby ruling out the possibility that persisting signals from the adapted eye contributed to these transfer effects.

In the third *Nature* paper to come out of his PhD work (Blake & Fox, 1974), Randolph once again took the psychoanatomical approach, devising a novel paradigm that evaluated the suppressive influence of binocular rivalry on the cortical mechanisms of visual adaptation. By psychophysically measuring adaptation to the suppressed stimulus, Blake and Fox were able to infer the extent to which this stimulus still finds a cortical representation and to thereby shed light on the relation between this representation and the process of interocular combination. Incidentally, the work also foreshadows, two decades early, an entire research tradition that examines psychophysical, as well as neural, measures under interocular suppression in the search for neural correlates and determinants of visual awareness. Randolph himself would also, at various points in his career, revisit the combination of visual adaptation and suppression to investigate topics as diverse as the neural basis of negative afterimages (Gilroy & Blake, 2005) and the phenomenon known as stimulus rivalry (Brascamp, Sohn, Lee, & Blake, 2013; Lee & Blake, 1999), the relation between attention and awareness (Alais & Blake, 1999; Brascamp, van Boxtel, Knapen, & Blake, 2010; Ling & Blake, 2012), and the perception of facial expressions (Yang, Hong, & Blake, 2010). In a characteristic display of intellectual honesty, he would also revisit his own 1974 findings and show that they represented a special case within a broader phenomenon carrying a substantially different interpretation (Blake, Tadin, Sobel, Chong, & Raissian, 2006).

 

**Karen Yu:** Randolph was a remarkable mentor. I remember working on manuscripts together, sitting side by side in front of the computer in his Wilson Hall office on the Vanderbilt campus. I remember the campus walk he invited me on a day or two before my dissertation defense and how, through our conversation and his thoughtful questions, he helped me raise my awareness of potential challenges and bolstered my confidence. And I'm especially grateful for how attuned and supportive he was to my shifting interests, away from a research-intensive path toward a teaching-focused one. He called my attention to small liberal arts colleges, and in particular to a rare tenure-track opening at a nearby hidden gem that I hadn't yet heard of—and it's thanks to him that I've had the joy of teaching for nearly 30 years now at the University of the South (Sewanee).

 

**Kevin Dieter:** One of the most impressive things about Randolph's mentorship in my view is his dedication to it. He was always willing to find a time to troubleshoot a test design or even come to the lab and run through the experiment himself and offer his feedback. Being able to learn from his thoughtful, rigorous problem-solving approach has helped me navigate a wide range of research and analytics organizations, and I am very grateful for all he has contributed to my career!

## Rivalry and cooperation

It would be difficult to overstate Randolph Blake's impact on the study of binocular rivalry. While the foundation was laid in his PhD and postdoctoral years, during his early faculty years at Northwestern University (1975–1988), Randolph truly transformed binocular rivalry from a perceptual curiosity into an instrument capable of probing the inner workings of the visual system. Through a systematic series of studies, he characterized the spatial scale of competitive interactions during rivalry suppression (Blake, O'Shea, & Mueller, 1992), the monocular specificity of this suppression (Blake, Westendorf, & Overton, 1980), and how the presence of stereoscopically fusible elements can override binocular conflict altogether (Blake & Boothroyd, 1985). Across these studies, a coherent picture began to emerge: Rivalry was not simply a case of failed binocular combination but instead the result of active competitive processes within early visual cortex. This insight culminated in “A Neural Theory of Binocular Rivalry” in *Psychological Review* (Blake, 1989)—a model that proposed reciprocal inhibition between orientation-tuned monocular V1 neurons as the mechanistic basis of rivalry, with adaptation governing the dynamics of dominance and suppression. This model, both parsimonious and testable, set the agenda for rivalry research for decades.

While that 1989 article effectively served as a summation of binocular rivalry work up to that point, the field was on the verge of major change. In the years that followed, binocular rivalry research underwent a rapid transformation, and those years highlight another major catalyst for Randolph's remarkable career: his collaborative spirit.

One factor that, in the 1990s, drove the transformation of binocular rivalry science, and of vision science as a whole, was the emergence of fMRI as a research tool, making the contents of the human brain accessible noninvasively. Randolph, who had returned to Vanderbilt University by then, recognized the general importance of fMRI and also its promise for the study of binocular rivalry. After all, his model had directly implicated early visual cortex in driving rivalry, and suddenly the field was armed with a method that could noninvasively vet these ideas. To pull this off, Randolph partnered with David Heeger, one of the pioneers of functional neuroimaging. Randolph and David's collaboration proved productive. Their first collaborative effort culminated in a landmark study in *Nature Neuroscience* (Polonsky, Blake, Braun, & Heeger, 2000), which revealed some of the first neural evidence in favor of low-level visual competition during rivalry. This study, along with other work that, around the same time, linked subjective perception to visuocortical activity (Tong, Nakayama, Vaughan, & Kanwisher, 1998; Tong & Engel, 2001), marked a watershed moment in the field of human vision science, helping to usher in a new wave of research.

Meanwhile, a debate was brewing—one that exemplifies Randolph's ability to not only join forces based on complementary strengths but also find common scientific ground amid diverse viewpoints. The 1990s saw the field's understanding of rivalry seemingly upended by a series of seminal neurophysiological studies by Nikos Logothetis and colleagues. These studies found neuronal evidence that rivalry competition occurred in higher extrastriate visual areas of awake-behaving monkeys, rather than at the early cortical stages implicated by, among others, Randolph's work (Logothetis & Schall, 1989; Leopold & Logothetis, 1996; Sheinberg & Logothetis, 1997). This led to a lively debate over whether binocular rivalry arises from a low-level form of interocular competition or higher-level forms of pattern competition. One could easily have started a potentially zero-sum scientific battle. But in his response, Randolph displayed a greater interest in advancing the field as a whole than in a protracted debate. Flexing his ability to find common ground, he opted for the path of collaboration and took this as an opportunity to propose and co-write a highly influential review article on binocular rivalry with Nikos Logothetis in *Nature Reviews Neuroscience* (Blake & Logothetis, 2002). This paper sublimated the debate into a remarkably accessible and thoughtful review in which the two authors adopt a conciliatory voice, while noting the talking points where they agree to disagree. It is no coincidence that this consensus-building paper is Randolph's most cited publication with over 1,800 citations. Clearly, the field recognized this work for what it was: not a concession, but an act of scientific leadership through cooperation.

 

**Duje Tadin:** As a first-year graduate student, I worked on teasing apart interactions between form and motion, testing whether perception of motion among independent elements would be affected by imposing a form structure on those elements (e.g., by organizing elements into an upright biological walker). We found that the overall form structure had considerable effects on how element motion was perceived. But it appeared to me that we were “scooped” by a paper in *Nature Neuroscience*. I was, needless to say, disappointed. Randolph's response was: Let's just put our “thinking hats” on and focus on the big picture. This is where Randolph suggested thinking about our work in a broader context of reference frames for encoding visual information, and we eventually wrote a paper entitled “What Constitutes an Efficient Reference Frame for Vision?” that was also published in *Nature Neuroscience*. This was an invaluable mentoring lesson for me (as a starting graduate student), where Randolph showed me the importance of “big picture” thinking and the power of intuition. Many of the big advances in science come from intuiting connections between different concepts and/or levels of analysis—this is an area where Randolph truly excels.

That same bridge-building tendency characterized Randolph's approach to computational modeling. Rather than treating empirical psychophysics and formal theory as separate enterprises, Randolph recognized them as natural counterparts and sought collaborations that could translate qualitative ideas about rivalry into quantitative models. One continuing thread throughout his career, in this regard, is his collaboration with Hugh Wilson. The products of this collaboration, while including a strong psychophysical component, also featured influential computational contributions. Perhaps the best illustration of this, as well as of Randolph's general ability to devise elegantly designed science through the marriage of different perspectives, is a stunning series of papers from the early 2000s. The first is 2001’s influential *Nature* piece “Dynamics of Traveling Waves in Visual Perception” by Wilson, Blake, and then-graduate student Sang-Hun Lee (Wilson, Blake, & Lee 2001). Starting from the observation, informally made by many researchers, that rivalry dominance tends to spread in a wave-like fashion across the visual field, this team devised a novel paradigm to contain this spread and measure its properties. The resulting paper spanned the full range from the initial subjective observation to precise psychophysical characterization, inferences about underlying neural dynamics, and, finally, quantitative modeling of these dynamics. This work was then followed by two elegant studies published in *Nature Neuroscience* (Lee, Blake, & Heeger, 2005; Lee, Blake, & Heeger, 2007), in which the team used fMRI to demonstrate that these perceptual traveling waves could be attributed to spatiotemporal modulations of activity that sweep across the early visual cortex.

 

**Eunice Yang:** Randolph Blake is as exceptional a human being as he is a scientist. His genuine excitement for science was contagious, and his mentorship was marked by the kind of thoughtful, compassionate guidance that shapes not just careers but people. Perhaps nothing captures his selflessness more than a singular gesture: Knowing I had never been to Korea and might not otherwise have the chance to go, he invited me to collaborate with researchers there—an experience that connected me not only to the work, but to my own heritage. That is Randolph: always thinking beyond the work and always thinking of others.

In the years that followed, Randolph continued forging collaborative efforts to settle contested questions in rivalry. For example, there had been a long-running debate about the role of frontal and parietal cortex in initiating rivalry alternations. Randolph and colleagues contributed to this debate, first, by pairing fMRI with inventive psychophysical designs to mount a challenge to the claim that top-down feedback from frontoparietal areas drives perceptual switching during rivalry. They showed that the extent of frontoparietal activity accompanying rivalry alternations scales with the alternations’ conspicuity (Knapen, Brascamp, Pearson, van Ee, & Blake, 2011) and reduces to zero when alternations are rendered inconspicuous to the point of being unreportable (Brascamp, Blake, & Knapen, 2015). Their conclusion was pointed: Frontoparietal areas are unlikely to initiate rivalry alternations per se; rather, competitive interactions within visual cortex itself most plausibly account for the perceptual alternations in rivalry and other forms of bistable perception. True to form, Randolph then followed up these research studies with a synthesizing review piece that included authors on both sides of the debate (Brascamp, Sterzer, Blake, & Knapen, 2018).

 

**Jan Brascamp:** Here's something that's striking to me. My time in Randolph's lab shaped me in many ways—how I try to write, how I go about scientific debate, how I approach mentoring, to name a few. Yet, when I try to recall times that Randolph told me how to do any of those things, I come up with exactly one—and the only reason I recall that time is probably that it was so unusual for him to instruct me at all. Randolph's method was not telling; it was showing. In his lab, you improved through a process of osmosis, by spending time close to someone whose ability was so obvious and who shared it so selflessly. Recently, getting (back) in touch with some of Randolph's other former mentees in the context of his Festschrift, I've started to suspect that the word “method” here is wrong—what I described above is less a professional approach than a way of being that Randolph brings to all aspects of life. Just as his mentees agree about all that they have learned from Randolph, there is also a uniform fondness for him and Elaine. That is curious to me because, just as Randolph taught mostly by example rather than instruction, he and Elaine seemed to form bonds with people indirectly. Rather than ever being overbearing, performative, or even especially forward, they made people feel welcome and cared for and, in the process, created precisely those connections around them.

## To boldly go

Our story so far has emphasized Randolph's work on binocular vision, rivalry in particular. Indeed, his name typically elicits strong associations with that work, and if his career had consisted of that work alone, it would be a career most of us can only marvel at. What is amazing, however, is how many contributions Randolph has made outside of this domain and how impactful those contributions are in their own right. The same mindset—characterized by rigor, collaborative spirit, and, perhaps above all, genuine love of the process of discovery—led him to successfully take on research in a diverse set of areas, as well as other endeavors. The breadth of his intellectual curiosity is equally matched by its depth. It is telling that, out of his three most cited papers, two are not about binocular vision at all but about the perception of biological motion (Blake & Shiffrar, 2007; Grossman et al., 2000). The latter is a highly influential review article, and the former is a pioneering fMRI study into the neural representation of biological motion, led by then-graduate student Emily Grossman, with whom Randolph would publish a series of further high-profile studies on the topic (Grossman & Blake, 2001; Grossman & Blake, 2002). Another illustration of Randolph's research output beyond binocular vision is his work on synesthesia. He was deeply involved in an early influential study that demonstrated the “Perceptual Reality of Synesthetic Colors” (Palmeri, Blake, Marois, Whetsell, & Flanery, 2002) and subsequently published multiple papers on this topic.

 

**Hideko Fukuda Norman:** When I first arrived at the Nashville airport from Japan as a visiting scholar, it was Randolph who picked me up and brought me to the apartment that had been arranged by the department for me to stay.  The very next day, he even brought me to Target and Kroger for shopping.   What a nice, generous, and helpful mentor!!

 

**Sam Ling:** Memories can beguile. You'd think it should be effortless for me to conjure a vivid mental image of what it was like to be there in Randolph's office, tucked in the corner of Wilson Hall. Those were, after all, some of my favorite moments as a scientist; surely, they should distill into a rich, episodic anecdote. But when I close my eyes and travel back, the events themselves—however formative—emerge as a blur. What remains instead are crystallized strands: the deafening sighs of cicadas, the afternoon light cast across the objects in his office. Randolph's shelves, adorned with not only science and textbooks, but rich in texture. Rows of identically shaped water jugs, each a different color, each intentionally placed. Those objects were a constant, quietly taking over as placeholders for my memories of that period—of working with Randolph, thinking alongside him. But one piece, in particular, sits in the foreground. A globelike sculpture—clay, perhaps—illuminated from within. Randolph was unafraid to hold uncertainty, unafraid to sit with it, and turn it over. Working with him, I'd find myself sitting there silently gazing at that sculpture. Even then, I wasn't sure what I was seeing inside it. Was it a face? An abstraction? And now, with time passing, its ambiguity deepens. Sometimes, when I find myself puzzling over a student's data in my own lab, I close my eyes to consider the possibilities, and that sculpture takes shape*. Think.*

But evidence of Randolph's indefatigable intellectual curiosity is not limited to traditional scientific research, and we would be remiss not to at least briefly mention his varied achievements outside that domain. There is, for example, Randolph's 2006 Ig Nobel Prize, shared with coauthors for a 1986 study that combined a three-pronged garden tool with auditory frequency analysis to understand the chilling effect of the sound of nails on a chalkboard (Halpern, Blake, & Hilenbrand, 1986). Moreover, together with Robert Sekuler, Randolph made a long-lasting contribution to vision science by writing and updating a widely adopted undergraduate textbook. This textbook on *Perception*, first published in 1985 (Sekuler & Blake, 1985), was continually revised over five editions spanning a period of 31 years, contributing to the education of generations of undergraduate students, and inspiring some of the very best students to pursue graduate studies in vision science. A different publication by the same author team, however, may be an even better illustration of Randolph's wonder-driven spirit. In 1998, they published the book *Star Trek on the Brain: Alien Minds, Human Minds*, which connects the fictional world of the TV franchise with real discoveries in psychology and neuroscience (Sekuler & Blake, 1998).

### In a nutshell . . .

Randolph Blake has had a profound influence. This is apparent from just a glance at his CV: over 300 published papers appearing in the field's top journals, amassing more than 35,000 citations and an H-index currently topping 90. Randolph's contributions span the full breadth of vision science, ranging from binocular vision to synesthesia, and from motion processing to the neural bases of visual awareness. Yet beneath this remarkable breadth lie a number of recurring themes that we have tried to trace throughout this retrospective. Taken together, they reveal not just the science but also the scientist: his way of seeing problems, his way of approaching them, and ultimately the cast of a mind that shaped a lifetime of discovery.

Through his vision and curiosity-driven approach to inquiry, Randolph cultivated a subsequent generation of scientists, all of whom carry forward this legacy. Perhaps the most telling sign of that influence: At Randolph's Festschrift, more than two dozen former trainees and collaborators flew in from across the world to celebrate his career in person (Figure 1). At that event, there was a palpable sense that we all had a shared, yet ineffable experience: *stumbling into his office, distraught by a thorny problem, but then emerging an hour later, energized, oriented, and ready to run the next experiment*. Randolph has a near-magical ability to open people's minds —through a combination of intellectual frankness, genuine curiosity, and a warmth and humility that puts even the most tangled thinker at ease. In that sense, Randolph Blake cultivated something that citation counts cannot easily capture: a spirit of curiosity, rigor, and genuine delight in the doing of science, one that his trainees propagate into their own work, labs, and classrooms every day.

**Figure 1.**
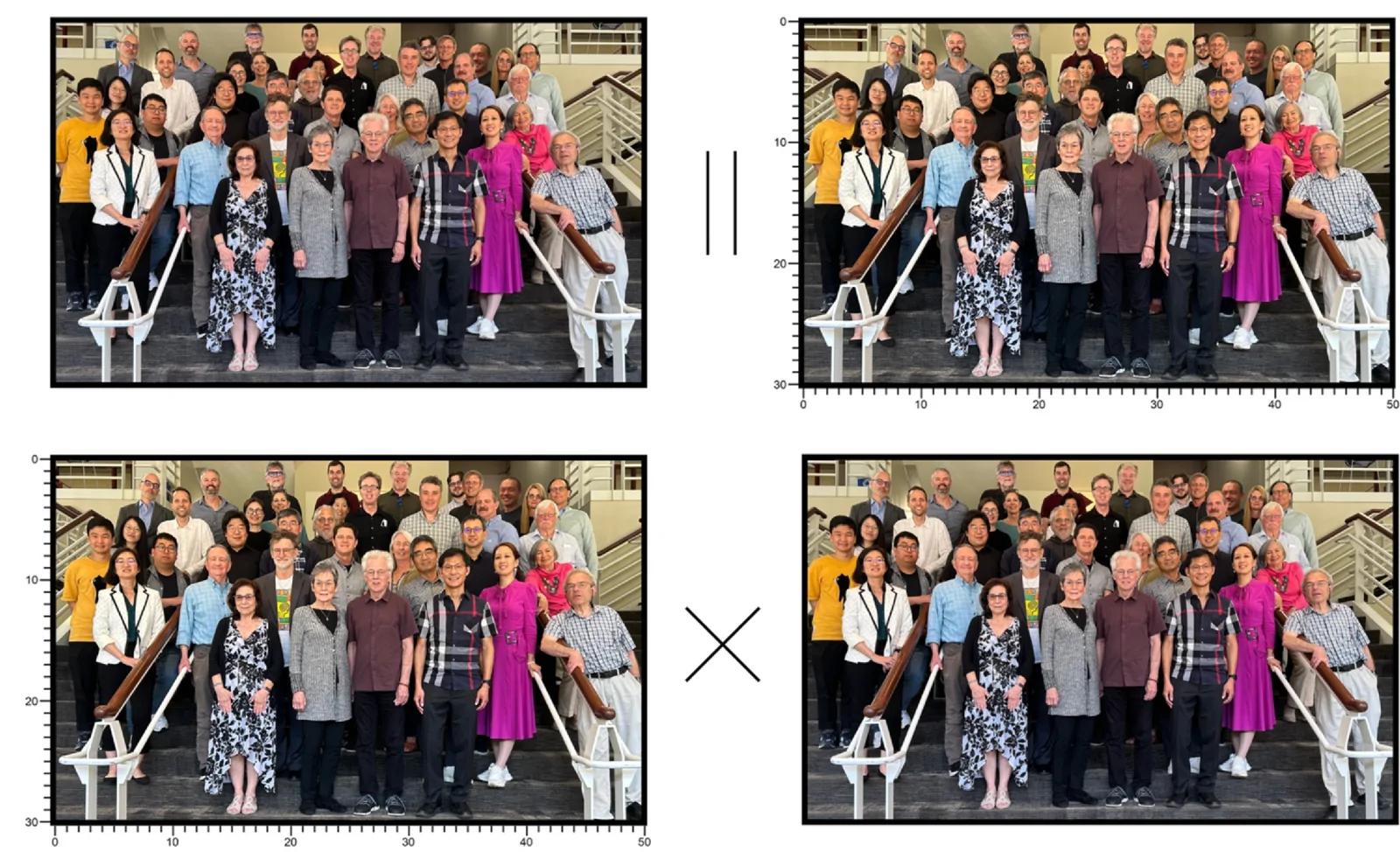
Stereo photograph showing attendees of Randolph Blake's Festschrift in the lobby of the Department of Psychology, Vanderbilt University (May 2024). Parallel fusers should foveate the top pair of images, cross-fusers the bottom pair. Attendees (numbers in parentheses are face coordinates as marked along the image edges): Karen Holopigian (16,12); Elaine Blake (22,11); Randolph Blake (27,10); Frank Tong (34,10); Jeremy Wolfe (45,9); Joclyn Sy (5,9); Jinhyeok Jeong (10,8); Joe Lappin (14,9); Robert O'Shea (20,8); David Alais (25,7); Emily Grossman (30,7); Sang Wook Hong (32,8); Chai-Youn Kim (39,8); Frances Wilkinson (42,8); Jeunghwan Choi, (3,6); Jisoo Sun (6,6); Oakyoon Cha (15,6); Min-Suk Kang (19,5); Tom Palmeri (22,5); Sang Chul Chong (35,6); Hugh Wilson (42,5); Jan Brascamp (11,4); Sohee Park (16,4); Connie McTigue, (18,3); Soo-Eun Chang (24,5); Rene Marois (26,2); Duje Tadin (31,3); Ken Sobel (38,4); Sam Ling (40,2); Michelle Schall (41,3); Jeff Schall (42,2); Geoff Hurst, (8,2); Tomas Knapen (13,2); Stephen McTigue (19,1); Kevin Dieter (23,1); Alexander Maier (28,1); Brock Carlson (33,2); Geoff Woodman (35,2).
